# Factors Predicting the Response to a Vitamin D-Fortified Milk in Healthy Postmenopausal Women

**DOI:** 10.3390/nu11112641

**Published:** 2019-11-04

**Authors:** Rebeca Reyes-Garcia, Antonia Garcia-Martin, Santiago Palacios, Nancy Salas, Nicolas Mendoza, Miguel Quesada-Charneco, Juristo Fonolla, Federico Lara-Villoslada, Manuel Muñoz-Torres

**Affiliations:** 1 Centro de Investigación Biomédica en Red sobre Fragilidad y Envejecimiento Saludable (CIBERFES), Instituto de Salud Carlos III, 28029 Madrid, Spain; rebeca.reyes.garcia@gmail.com (R.R.-G.); garciamartin_t@hmail.com (A.G.-M.); 2 Unidad de Endocrinología y Nutrición. Hospital Universitario Torrecárdenas, 04009 Almería, Spain; 3 Unidad de Gestión Clínica Endocrinología y Nutrición, Hospital Universitario San Cecilio de Granada, Avenida de la Innovacion, 18016 Granada, Spain; charneco@me.com; 4 Palacios Institute of Women’s Health, 28029 Madrid, Spain; santiago.palacios@institutopalacios.com (S.P.); nancy.salas@institutopalacios.com (N.S.); 5 Department of Obstetrics and Gynecology, University of Granada, 18016 Granada, Spain; NICOMENDOZA@telefonica.net; 6 Nutrition Department, Biosearch S.A, 18016 Granada, Spain; juristo.fonollajoya@biosearchlife.com; 7 Research and Development Department of Lactalis Puleva, Nutrition Department, Biosearch S.A, 18004 Granada, Spain; federico.laravilloslada@puleva.es; 8 Department of Medicine, University of Granada, 18016 Granada, Spain; 9 Instituto de Investigación Biosanitaria (Ibs.GRANADA), 18106 Granada, Spain

**Keywords:** Vitamin D, postmenopausal women, obesity, fat mass

## Abstract

Background: Milk products fortified with vitamin D may constitute an alternative to pharmacological supplements for reaching the optimal levels of serum 25-hydroxyvitamin D [25(OH)D]. Our aim was to analyze the response of serum 25(OH)D and its predictive factors in postmenopausal healthy women after a dietary intervention with a milk fortified with vitamin D and calcium. Methods: We designed a prospective study including 305 healthy postmenopausal women who consumed a fortified milk with calcium (900 mg/500 mL) and vitamin D3 (600 IU/500 mL) daily for 24 months. Results: The 25(OH)D concentrations at 24 months were correlated to weight, to body mass index, to the percentage of fat, triglycerides and to baseline 25(OH)D levels. We found significant differences in the levels of 25(OH)D at 24 months according to baseline 25(OH)D levels (*p* < 0.001) and body mass index (*p* = 0.019) expressed at quartiles. Multivariate analysis showed an association between levels of 25(OH)D after the intervention and at baseline 25(OH)D (Beta = 0.47, *p* < 0.001) and percentage of body fat (Beta = −0.227, *p* = 0.049), regardless of the body mass index. Conclusions: In healthy postmenopausal women, the improvement in 25(OH)D after an intervention with a fortified milk for 24 months depends mainly on the baseline levels of serum 25(OH)D and on the percentage of body fat.

## 1. Introduction 

The Institute of Medicine (IOM) states that the Recommended Dietary Allowance (RDA) of vitamin D is 15 μg (600 IU, international units) for 97.5% of the population aged 1–70, and 20 μg (800 IU) for 97.5% of the population >70 years. These are the recommendations for achieving the circulating levels of 25-hydroxyvitamin D (25(OH)D) ≥ 20 ng/mL needed to maintain bone health [1]. However, other guidelines recommend serum levels of at least 20–30 ng/mL [2,3], and according to these recommendations, many people are vitamin D deficient and will need vitamin D supplementation [4]. However, there is an important controversy about the target serum levels of 25(OH)D that must be reached to achieve the maximum health benefits and how to supplement 25(OH)D in cases where it is indicated [5].

The consumption of foods fortified with vitamin D is an alternative to treatment with pharmacological supplements to reach the optimal serum levels of 25(OH)D. Factors influencing the response to this strategy constitute an interesting area of research to optimize the nutritional recommendations about fortified foods. Although there is no consensus, several clinical factors have been reported to influence the dose–response relationships between vitamin D supplementation and serum 25(OH)D [6,7], such as body weight, percentage of fat, age, baseline 25(OH)D levels, and type and the duration of the intervention. In addition, genetic factors like single nucleotide polymorphisms in the vitamin D-binding protein gene can also be significant [8]. 

Obesity is one of the main factors related to a lower response after vitamin D supplementation [7]. Different causes have been proposed to explain this finding, one of them is a decreased bioavailability of vitamin D_3_ from skin and from dietary source due to its deposition in body fat compartments. Also, an increased distribution volume for vitamin D has been proposed. Vitamin D deficiency is related to obesity regardless of age and the latitude, and it is also independent of the cut-offs to define vitamin D deficiency [9]. In the context of the increasing prevalence of obesity in the worldwide population, a better knowledge of the relationship between obesity and vitamin D after nutritional interventions is of interest. 

Therefore, the aim of our study was to evaluate the changes occurring in serum 25(OH)D levels and their predictive factors in postmenopausal Spanish women after a nutritional intervention with a dairy product fortified with vitamin D.

## 2. Materials and Methods

### 2.1. Study Design 

The findings presented in this study are a post-hoc analysis of data from a previously published clinical trial [10]. Here we analyze the predictive factors of response of vitamin D after 24 months of a nutritional intervention with a fortified milk with calcium (900 mg/500 mL) and vitamin D3 (600 IU/500 mL) on serum 25(OH)D. For this analysis, we selected 305 postmenopausal healthy women (mean age 59 ± 6 years) who completed 24 months of follow up. Inclusion and exclusion criteria of the study have been previously published [10]. 

Women consumed the dairy drinks for 24 months (500 mL/day, two intakes per day of 250 mL each), in the context of their usual diet. Counselling about Mediterranean diet and physical activity were provided to all women. The adherence to the intervention was evaluated every three months by telephone calls and empty dairy containers were collected. Compliance with the intervention was above 90%. The dairy drinks were produced in white 1 L Tetra Bricks by Lactalis Puleva (Granada, Spain). 

The study was approved by the Ethics Committee of Hospital Universitario San Cecilio of Granada. All the volunteers provided informed written consent. The study was conducted in accordance with the ethical principles of the Declaration of Helsinki, following the EEC Good Clinical Practice guidelines (July 1996).

### 2.2. Anthropometric Measurements

In the first visit anthropometric measurements were obtained, and also after 24 months of intervention. We measured body weight (kg) using a standard balance beam scale (Seca), and body height (cm) using a precision stadiometer (Seca), attached to the balance beam scale. Obesity was defined as body mass index >30 kg/m^2^. 

### 2.3. Body Composition Measurements 

Skeletal muscle mass was estimated from bioelectrical impedance analysis (Tanita BC418) at baseline and at 24 months after the onset of the intervention. This device calculates the percentage of body fat, fat mass, fat-free mass, and the predicted muscle mass, based on the data obtained by Dual-Energy X-ray Absorptiometry (DXA), using Bioelectrical Impedance Analysis with an operating frequency of 50 kHz at 500 lA. Obesity was defined as percentage of fat mass above 35% [11]. 

### 2.4. Biochemical Parameters

Blood samples were obtained at 0 and at 24 months after a 12 hour overnight fast. The blood was collected in a SST-Vacutainer (BD) and serum was separated by centrifugation at 3000 rpm for 15 min at 22–24 ℃, then it was divided into aliquots and frozen and stored at −80 ℃ until analysis. Serum total cholesterol, high-density lipoprotein cholesterol (HDL-c), triglycerides (TGs), glucose levels, HbA_1c_, and apo B were determined by standard automated procedures (Biosystems, Barcelona, Spain). LDL-c was calculated using the Friedewald formula. Serum 25(OH)D levels were measured by chemiluminescence immunoassay from Diasorin (LIAISON^®^ 25 OH Vitamin D TOTAL Assay). 

### 2.5. Statistical Analyses

Data were evaluated for normality and homogeneity of variance, and they are expressed as mean standard ± deviation. The relationship between serum vitamin D and biochemical and clinical factors were assessed by univariate analysis. A logistic regression and multiple regression analysis were performed to analyze the association between vitamin D levels at baseline and the levels found after 24 months of intervention. The variables related to vitamin D after 24 months of intervention (percentage of fat mass, weight, BMI, triglycerides and baseline 25 OH vitamin D) were included as covariates. SPSS software (version 17.0, IBM, Armonk, NY, USA) was used for doing statistical analysis. We considered *p* values < 0.05 as significant.

## 3. Results

Baseline clinical characteristics are shown in Table 1. Mean age was 59.3 ± 5.9 years. 

### 3.1. Changes in Biochemical Parameters

Serum 25(OH)D concentrations increased significantly at 24 months compared to the baseline values (25.4 ± 6.3 ng/dL vs. 21.7 ± 8.3 ng/dL, respectively, *p* < 0.001). At baseline, 51.3% of the women had 25(OH)D concentrations > 20 ng/mL, and 14.1% > 30 ng/mL. After 24 months of intervention, we observed a significant increase in the percentage of women with 25(OH)D levels > 20 ng/mL (78.5%) and > 30 ng/mL (18.8%) compared to baseline, *p* < 0.001 for both.

### 3.2. Factors Related to 25(OH)D Levels after the Intervention

**Figure 1:**
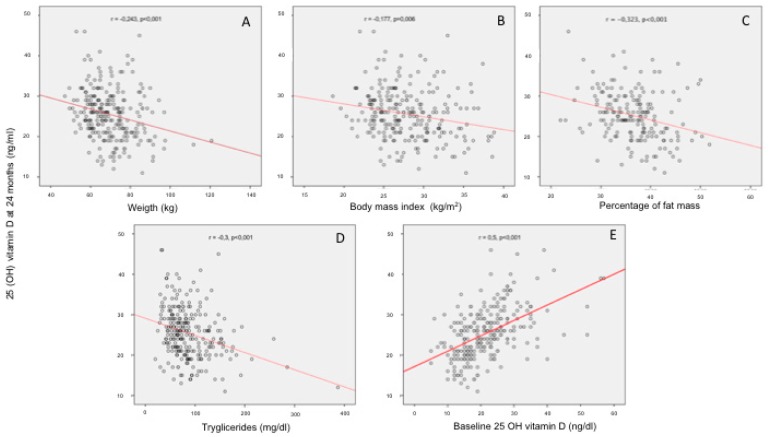
Correlations between serum 25(OH)D levels at 24 months and weight (**A**), body mass index (**B**), percentage of fat mass (**C**), triglycerides (**D**) and baseline 25(OH)D levels (**E**).

**Figure 2:**
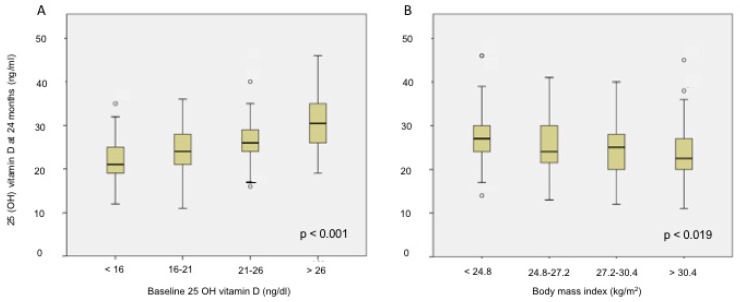
The 25(OH) D levels at 24 months according to quartiles of baseline 25(OH) vitamin D (**A**) and quartiles of body mass index (**B**).

Serum 25(OH)D levels at 24 months were correlated to weight (*r* = −0.243, *p* < 0.001), BMI (*r* = −0.177, *p* = 0.006), percentage of fat mass (*r* = −0.32, *p* < 0.001), triglycerides (*r* = −0.301, *p* < 0.001) and baseline 25(OH)D levels (*r* = 0.5, *p* < 0.001). (Figure 1). We found statistically significant differences in the levels of 25(OH)D at 24 months according to baseline 25(OH)D (*p* < 0.001) and BMI (*p* = 0.019) distributed by quartiles (Figure 2). 

We did not find differences in baseline 25(OH)D according to BMI: (<30 kg/m^2^) 21.9 ± 8 vs. (>30 kg/m^2^) 21.3 ± 8.5, *p* > 0.05. However, lower 25(OH)D levels were observed in women with BMI > 30 kg/m^2^ (24.1 ± 6.5 ng/mL) vs. those women with a BMI < 30 kg/m^2^ (26.2 ± 6 ng/mL) at 24 months (*p* = 0.026). 

In women with obesity, defined by percentage of fat mass, baseline levels of 25(OH)D were lower (21.2 ± 8.1 ng/mL) compared to women with percentage of fat mass below 35%: (24.6 ± 9 ng/mL), *p* < 0.01. Women with obesity, defined by percentage of fat mass, also reached lower 25(OH)D levels after 24 months of intervention: 23.7 ± 5.9 ng/mL vs. 27.4 ± 5.9 ng/mL, *p* < 0.001, compared to women with percentage of fat mass below 35%. 

### 3.3. Multivariate Analysis

When we analyzed the probability of reaching adequate vitamin D levels after 24 months of intervention, women with obesity (defined as BMI > 30 kg/m^2^ or percentage of fat mass above 35%) have a higher risk of 25(OH)D < 20 ng/mL at 24 months: odds ratio (OR) 2.3, confidence interval, CI, 95%: 1.2–4.4, *p* = 0.013 for BMI > 30 and OR 5, CI 95% 2–12.6, *p* < 0.001, for percentage of fat mass. The influence of adiposity, defined as percentage of fat mass, in 25(OH)D levels after 24 months persisted after adjusting for BMI: 25(OH)D < 20 ng/mL OR 4.5 CI 95% 1.6–12.3, *p* = 0.003, 25(OH)D < 30 ng/mL OR 3.2 CI 95% 1.2–8.9, *p* = 0.02.

In women with percentage of fat mass above 35%, there was a 3.2 times higher probability of reaching 25(OH)D levels < 30 ng/dL at 24 months, regardless of the BMI. However, when we adjusted by baseline 25(OH)D levels, only the relationship with 25(OH)D < 20 ng/dL persisted (OR 3.6, CI 1.3–10.1, *p* = 0.007).

In the multivariate analysis, we observed an association between serum 25(OH)D after 24 months of intervention, baseline 25(OH)D levels (Beta = 0.47, *p* <0.001) and percentage of fat mass (Beta = −0.227, *p* = 0.049), regardless of weight, BMI and triglycerides.

## 4. Discussion 

The daily consumption of a dairy product providing 600 IU of vitamin D3 for 24 months is effective to improve serum concentrations of 25(OH)D in healthy postmenopausal women. The nutritional intervention described in our study allows that 78.5% of women reach 25(OH)D levels > 20 ng/mL, and 18.8% of women reach 25(OH)D > 30 ng/mL. Baseline 25(OH)D levels and the percentage of body fat mass are the main factors explaining the responsiveness. The risk of having 25(OH)D below 20 ng/mL after 24 months of intervention was 3.6 times higher in the women with fat mass above 35%, regardless of BMI and baseline 25(OH)D levels. 

This study shows that a simple nutritional intervention with a vitamin D3-enriched milk that supplies 600 IU/day is effective in increasing 25(OH)D levels and helps to reach adequate 25(OH)D levels in a high percentage of women. These results are comparable to those described when pharmacological supplements of vitamin D were used, which often are not well tolerated by patients, especially if combined with calcium [12]. Although there is not a total consistency regarding the adequate 25(OH)D levels in healthy subjects [1,2,3,13], the most accepted serum values are 20 ng/mL, which will be reached for most of the women after this intervention. 

Basel concentrations of 25(OH)D influence the achievement of adequate 25(OH)D levels after 24 months, regardless of other factors. This fact reinforces previous data found in older [14,15] and younger adults [16], and confirms the importance of considering baseline vitamin D in postmenopausal healthy women who receive a nutritional intervention with vitamin D. 

Another independent predictor of the response of 25(OH)D levels after 24 months of intervention was body fat. Although weight, triglycerides and BMI showed and association with 25(OH)D levels at 24 months, only the percentage of fat mass persisted in multivariate analysis. BMI is a simple and worldwide measurement of obesity. However, its validity has been discussed in recent years [17], and other measures of fat may provide a better estimation of obesity, as percentage of fat mass. In the present study, only the percentage of fat mass remained as an independent factor influencing the response of vitamin D after the nutritional intervention, and not BMI. In older healthy women, fat mass was negatively related to 25(OH)D in a cross-sectional study [18]. Our data showing an independent association between the fat mass percentage and the evolution of 25(OH) D after 24 months confirms this relationship, and reinforces the utility of this measurement when addressing the response to an intervention with vitamin D. Other authors [16] found that body fat mass and BMI were not related the 25(OH)D response, speculating that total body mass instead than fat mass may determine the 25(OH)D response. The disparity in the results may be explained by the duration of the intervention and also by the mean BMI of the subjects included in the study. 

There is no agreement regarding why obesity would affect serum 25(OH)D levels. This influence could be explained by a reduced intake of vitamin D, a reduced sun exposure, an increased storage and/or sequestration of vitamin D in the adipose tissue. A volumetric dilution due to the distribution of 25(OH)D in larger fat volumes [6,7,19,20]. In addition, vitamin D stored in the adipose tissue may be less available for hydroxylation [14,16]. Therefore, if the increased prevalence of obesity worldwide is the explanation for the high rates of hypovitaminosis D reported is a matter that should be investigated. However, our study could not demonstrate a favorable effect of the consumption of a dairy product supplemented with vitamin D on the loss of body weight or fat mass. This finding is consistent with previously described findings [21].

Although BMI is the most widely used method to evaluate the presence of overweight and obesity, it has been criticized because BMI does not always reflect true body fatness, that may be better evaluated by the assessment of body fat and fat-free mass [22]. Bioelectrical impedance analysis is considered as the simplest, most reproducible and least expensive method for the evaluation of body composition in clinical practice. It has shown a high accuracy and an excellent correlation with DXA when assessing the percentage of body fat [23]. Therefore, bioelectrical impedance is cost-effective and feasible, and may replace DXA in assessing the body composition. Our results highlight the usefulness of bioelectrical impedance analysis in the evaluation of healthy postmenopausal women. This analysis may be useful in the selection of women at higher risk of vitamin D deficiency or in the selection of women who need a higher supplementation of a more frequent evaluation of vitamin D levels.

Our results show that, in healthy postmenopausal women, baseline 25(OH)D levels and obesity, defined as the percentage of fat mass, are independent determinants of 25(OH)D levels after 24 months of a nutritional intervention, and they are of clinical relevance. These findings may allow a better personalization of the supplementation with vitamin D for reaching adequate levels after the intervention. Moreover, it has been described that the response to vitamin D may differ according to the dose of vitamin D and the duration of the intervention [24]. Furthermore, the factors influencing this response may differ between pharmacological and nutritional supplementation, and this potential difference must be addressed. Considering our findings, the recommended vitamin D dose may be adapted to baseline vitamin D levels. In addition, the estimation of the percentage of fat mass may be better for predicting the response to nutritional interventions with vitamin D. 

Our study has several strengths, as the long follow-up for a nutritional intervention, and the exhaustive evaluation of compliance with the intervention. Limitations of the study are the absence of a comprehensive assessment of dietary intake of vitamin D, which may influence the results. However, women were advised not to change their lifestyle habits, and exercise was evaluated semi-quantitatively and showed no changes during the intervention, as an example of no changes. In addition, we evaluated Caucasian healthy women, and the generalization of this finding to males or to other ethnic groups must be confirmed. Moreover, we only measured total 25(OH)D, and we did not evaluate other metabolites. 

## 5. Conclusions

In summary, the response of serum 25(OH)D levels to the supplementation with a fortified milk consumed for 24 months depend on baseline levels of serum 25(OH)D and on the percentage of body fat, regardless of BMI, in healthy postmenopausal women. In these women, the determination of the percentage of fat mass by bioelectrical impedance may allow a better prediction of the response to vitamin D after a nutritional intervention. 

## Figures and Tables

**Table 1:** Characteristics of study subjects at baseline and after 24 months of intervention.

	Baseline	24 months	*p*
Weight (kg)	70 ± 11	70 ± 11	0.5
Body mass index (kg/m^2^)	28 ± 4	28 ± 4	0.084
Obesity (%)	28	32	
Overweight (%)	47	48	
Percentage of fat mass	37 ± 5	37 ± 5	0.047
Serum calcium (mg/dL)	9.7 ± 0.6	9.8 ± 0.6	<0.001
Parathyroid hormone (pg/mL)	57 ± 21	58 ± 20	0.102
25(OH)D (ng/mL)	22 ± 8	25± 6	<0.001
Total cholesterol (mg/dL)	214 ± 33	208 ± 32	<0.001
HDL colesterol (mg/dL)	55 ± 15	55 ± 14	0.62
LDL colesterol (mg/dL)	141 ± 32	135 ± 32	<0.001
Triglycerides (mg/dL)	87 ± 44	88 ± 46	0.6
